# P2X receptors and lipids interact to modulate inflammation

**DOI:** 10.1016/j.jlr.2026.101084

**Published:** 2026-06-19

**Authors:** Vitor Nascimento Vidal, Thalita Calvet Pereira, Guilherme Pegas Teixeira, Leandro Rocha, Robson Xavier Faria

**Affiliations:** 1 Postgraduate Program in Plant Biotechnology and Bioprocesses, Center of Health Sciences, Federal University of Rio de Janeiro, Rio de Janeiro, RJ, Brazil; 2 Environmental Health Assessment and Promotion Laboratory, Oswaldo Cruz Institute, Rio de Janeiro, RJ, Brazil; 3 Natural Products Technology Laboratory, Federal Fluminense University, Niteroi, RJ, Brazil

**Keywords:** P2X receptor, bioactive lipids, ATP, inflammation

## Abstract

P2X receptors are purinergic receptors activated in the presence of extracellular ATP. These receptors are involved in the pathogenesis of various diseases, including inflammatory diseases, and are also associated with pain. These ionotropic receptors are activated when ATP is released during the inflammatory process. This can occur because, during the inflammatory process, phospholipase A_2_ is activated, releasing arachidonic acid, which is converted by COX-1 and COX-2 into prostaglandins or thromboxanes. In this context, prostaglandins sensitize cells, leading them to release ATP, which binds to P2X receptors, thereby activating their ion channels. These channels can act in various ways: P2X_4_ is involved in neuropathic pain, whereas P2X_7_ receptors can activate the NLRP3 inflammasome, which, in turn, induces IL-1β release and activates the COX pathway, generating an inflammatory cycle. In addition to prostaglandins, other bioactive lipids are also associated with increased expression of P2X receptors, such as leukotrienes and thromboxanes, which can increase Ca^2+^ influx, facilitating the release of extracellular ATP. Bioactive lipids, such as resolvins, lipoxins, maresins, and protectins, which have anti-inflammatory effects and promote homeostasis, can attenuate inflammation. They act indirectly on P2X receptors by controlling the inflammatory process, thereby decreasing ATP release and lowering P2X receptor expression. There are also lipids that structurally assist in P2X receptor activity, such as cholesterol and ceramides. Ceramides also play a role in the cell signaling process, inducing apoptosis. This review clarifies mechanisms of interaction between purinergic receptors P2X family and bioactive lipids, therefore modulating the inflammatory response.

### P2X receptors

The ionotropic receptors of the P2X family are a group of 7 purinergic receptors that are activated in the presence of extracellular adenosine 5′-triphosphate (ATP). They are divided into P2X_1_, P2X_2_, P2X_3_, P2X_4_, P2X_5_, P2X_6_, and P2X_7_. When activated by extracellular ATP, P2X receptors open ion channels in the plasma membrane of various cell types (1). The 7 subtypes of P2X have several common characteristics, such as the passage of Na^+^, K^+^, and Ca^2+^ cations and two transmembrane helices (TM1 and TM2), in addition to having N- and C-termini, which perform regulatory functions in the receptors, such as sensitivity to ATP and pore opening time (2). P2X_7_ stands out for its long C-terminal tail, which is essential for the formation of a pore with high conductance, in addition to the activation of the inflammasome (3).

Among the 7 P2X subtypes, P2X_1_ plays a notable role in smooth muscle contraction and is highly expressed in arteries, arterioles, and the male reproductive tract. Because this subtype is highly expressed in the vas deferens, some studies suggest P2X_1_ as a therapeutic target for male contraceptives (4). P2X_1_ receptors are also expressed in immune cells, such as neutrophils, and play a role in platelet secretion and aggregation. Therefore, blockade of this receptor could reduce thrombus formation and decrease the release of proinflammatory factors (5). P2X_2_ and P2X_3_ receptors are easily found in cells of the nervous system, especially in sensory neurons. They play a crucial role in signal transmission because when activated by ATP, the receptor induces depolarization in sensory neurons (6). These receptors are also present in the afferent fibers of the carotid body and are important for detecting hypoxia in the carotid body. A study published in 2003 revealed that the absence of P2X_2_ in rats reduced ventilation in response to hypoxia, revealing the relevance of this receptor for detecting O_2_ levels (7). Because P2X_3_ is widely expressed in peripheral nerves and is associated with nociception and chemoreception, this subtype has emerged as an excellent therapeutic target for the treatment of chronic pain (8). P2X_4_ is the receptor of the P2X family that has the highest permeability to Ca^2+^; this subtype is expressed in different cell types, such as immune cells and nervous system cells. This receptor also participates in the inflammatory process, in addition to being implicated in the pain process (9). P2X_4_ is also involved in the allergic process: when activated in mast cells in the presence of ATP, it can increase IgE-induced degranulation and Syk phosphorylation, suggesting that this receptor may be a therapeutic target for allergic reactions (10). Like other receptors, P2X_5_ is involved in the inflammatory process and in the maturation of osteoclasts. When the P2X_5_ receptor is activated by ATP, the release of IL-1β occurs, which is a mechanism through which this subtype increases the production of osteoclasts. Therefore, P2X_5_ is an essential regulator of bone cells during the inflammatory process (11). A study has also shown that mice lacking P2X_5_ exhibit glucose tolerance, as the P2X_5_ receptor present in brown adipocytes modulates glucose metabolism and thermogenic gene expression (12). Unlike other P2X receptors, P2X_6_ does not rely solely on ionic channels; its primary function is to modulate other P2X receptors. Some P2X_6_ receptors remain intracellular, thereby stabilizing other receptors (13, 14). P2X_7_ is the primary purinergic receptor involved in the inflammatory process; consequently, P2X_7_ is associated with several diseases, including cancer and depression (15). When activated by ATP, P2X_7_ can release proinflammatory cytokines such as IL-1β. In addition, the P2X_7_ receptor is involved in cell death and the activation of the NLRP3 inflammasome, a pathway through which cells can release cytokines into the external environment, intensifying the inflammatory process and ultimately generating more extracellular ATP, which ultimately activates P2X_7_ again. This overexpression of the P2X_7_ receptor is related to several chronic diseases, such as rheumatoid arthritis (16, 17).

### Bioactive lipids

Polyunsaturated fatty acids (PUFAs) are a fundamental class of biologically active lipids that play a central role in inflammatory biology and serve as precursors of a wide range of lipid mediators that regulate both the amplification and resolution of the inflammatory response. In addition to their functional relevance, these fatty acids are essential nutrients because the human body cannot synthesize them in sufficient quantities, and diet is their primary source (18). Among the PUFAs of greater physiological importance, short-chain essential fatty acids, such as α-linolenic acid (ALA, omega-3) and linoleic acid (LA, omega-6), whose classification is based on the position of the first double bond, stand out. After ingestion, these compounds are metabolized via denaturation and elongation reactions, yielding biologically more active long-chain PUFAs, such as eicosapentaenoic acid (EPA), docosahexaenoic acid (DHA), and arachidonic acid (AA). These derivatives have greater functional effects, particularly in the context of inflammation and immune responses (19, 20, 21). The long-chain PUFAs formed are incorporated into cell membranes at the n-2 position of phospholipid molecules and stored as a metabolic reservoir. In response to cellular activation, including inflammatory, infectious, or immunological stimuli, these fatty acids are released by phospholipase A_2_ (PLA_2_), an enzyme widely recognized as central to initiating the inflammatory lipid signaling cascade (22, 23). After they are released into the cytosol, AA, EPA, and DHA become substrates for distinct metabolic pathways, particularly those involving cyclooxygenase (COX), lipoxygenase (LOX), and cytochrome P450 (CYP450), which are responsible for the formation of a wide variety of bioactive lipids (24, 25).

The cyclooxygenase pathway is mediated by the COX-1 and COX-2 isoforms, in which arachidonic acid is converted to prostaglandin H_2_ (PGH_2_), which then gives rise to prostaglandins and thromboxanes. These mediators are classically associated with inflammatory symptoms, such as vasodilation, increased vascular permeability, pain, fever, and platelet aggregation (26). The lipoxygenase pathway, from AA, promotes the formation of mediators such as leukotrienes, lipoxins, and other oxylipins. 5-LOX produces leukotrienes, which are involved in leukocyte recruitment and bronchoconstriction (27). On the other hand, the enzymes 12-LOX and 15-LOX catalyze the formation of lipoxins, which are associated with anti-inflammatory and proproblem-solving effects. In addition, when EPA and DHA are metabolized via this pathway, proresolving mediators, such as resolvins, protectins, and maresins, are formed, which contributes to the control of the inflammatory environment (28). Additionally, the CYP450 pathway is an alternative metabolic pathway for PUFAs, with AA derivatives participating in the regulation of the inflammatory response (29).

## Materials and methods

This review was conducted through a structured literature search to identify scientific evidence on the interaction between bioactive lipids and P2X purinergic receptors in inflammatory processes. Searches were performed for articles, dissertations, and theses indexed in major electronic databases such as the PubMed, Scopus, Web of Science, SciELO, and Google Scholar databases, including studies published up to February 2026. The search strategy used controlled descriptors and free terms combined with Boolean operators (AND and OR), including the keywords “P2X receptors”, “P2X_7_ receptor”, “bioactive lipids”, “lipid mediators”, and “inflammation”. Additional terms related to specific lipid classes, such as prostaglandins, leukotrienes, and others, were considered during a screening manual to ensure the comprehensiveness of the lipid mediators described in this review. Studies investigating molecular, cellular, or physiological mechanisms related to lipid-mediated modulation of P2X receptors under inflammatory conditions were included. Duplicate articles, editorials, abstracts without full text, and studies unrelated to purinergic signaling or inflammation were excluded. The selection was performed by screening titles and abstracts and reading the full text, with data regarding receptor subtype, lipid class, experimental model, detection pathways, and inflammatory effects.

## Results

### Lysophosphatidic acid

Lysophosphatidic acid (LPA) is a simple phospholipid that acts as a potent signaling molecule. LPA has approximately 6 G protein-coupled receptors, called LPAR1--6, which play important roles in processes such as cell proliferation, RHO activation, and Ca^2+^ mobilization. Furthermore, this phospholipid is involved in pathologies such as neuropathic pain, neurodegenerative diseases, and cancer (30).

In a previous study, LPA induced ATP release in primary rat microglial cultures via the LPA3 receptor, possibly via the activation of P2X receptors (31). These findings were also confirmed by microglial activation experiments, in which LPA_2_ receptor activation triggered the release of purines into the extracellular medium, thereby activating P2X_7_ in oligodendrocytes under in vitro conditions (32). Interestingly, ATP mediates bebbling formation in the plasma membrane of osteoblasts via an LPA-dependent pathway. In this study, the authors proposed that ATP- or BzATP-induced P2X_7_ receptor activation stimulates phospholipase D and A_2_ production. Consequently, these phospholipases generate LPA, which activates Rho-associated kinase, thereby causing the formation of blebbing (33). Furthermore, this P2X_7_/LPA signaling axis, combined with eicasonoids, plays a crucial role in skeletal remodeling and mechanotransduction (34).

P2X_3_ ion channels are also involved in LPA signaling. In a study by Wu *et al.*, the authors demonstrated that LPA and its receptor, LPAR1, which acts through the Rho-ROCK pathway, regulate P2X_3_ during the development of mechanical and spontaneous pain in bone cancer and that LPA increases P2X_3_ receptor expression (35). Recent experiments have shown that treating rat DRG neurons with LPA increases the functional activity of α,β-methylene-ATP at P2X_3_ receptors in a concentration-dependent manner. Further investigation revealed that LPA1 receptors, Gq/11 proteins, and the PKC signaling pathway mediated this modulation. Furthermore, LPA potentiation promotes P2X_3_ sensitization, thereby increasing the nociceptive effects evoked by α,β-meATP in rats (36).

### Prostaglandins

Prostaglandins are prostanoid derivatives of arachidonic acid that act primarily in the inflammatory process. These lipid agents are synthesized from arachidonic acid by phospholipase A_2_, enter the cyclooxygenase (COX) signaling pathway, and are converted into terminal prostaglandins. Prostaglandins include PGD2, PGE2, PGF2α, and PGI2. Their receptors are divided into 4 subtypes: EP1, EP2, EP3, and EP4 (37, 38).

A study revealed that the application of ATP to cultured dorsal root ganglion (DRG) neurons increased the level of cytosolic phospholipase A_2_ phosphorylated at Ser505 and caused the translocation of this cytosolic phospholipase A_2_ phosphorylated at Ser505 to the plasma membrane. This effect was neutralized by A-317491, a selective inhibitor of P2X_3_R/P2X_2/3_R receptors, suggesting that phospholipase A_2_ translocation is involved in this process (39). Previous data indicate that PGE2-induced hyperalgesia in an inflammatory process model requires prior ATP release and activation of the neuronal P2X_3_ receptor (40). Both homeopathic P2X_3_ and heteromeric P2X_2/3_ receptors are involved in nerve injury, with their expression increasing in sensory neurons (41). Interestingly, PGE2 promotes ATP-mediated ATPase activity and its analog, α,β-meATP, through the P2X_3_ receptor in dorsal root ganglion (DRG) neurons but not through heteromeric P2X_2/3_ receptors. This mechanism depends on the action of the EP3 receptor, to which PGE2 binds, thus activating the cAMP/PKA signaling cascade and increasing P2X_3_ expression, consequently promoting allodynia and hyperalgesia (42). Under inflammatory conditions in vivo, PGE2 binds to EP3 receptors, thus activating cAMP and Epac, which, in turn, activate the PKC signaling pathway. This signaling potentiates P2X_3_ receptor-mediated responses to PGE2, thus exacerbating hyperalgesia (43). In another study, Kunori and colleagues reported that α-meATP- and PGF2α-induced allodynia disappeared in mice lacking PGF2α receptors, indicating that α-meATP-induced allodynia is mediated by PGF2α receptors. Immunostaining for β-galactosidase revealed that PGF2α receptors colocalize with P2X_2_ and P2X_3_ receptors in spinal cord neurons. These data indicate that PGF2α is involved in the mechanism of α-methylene ATP-evoked allodynia, with P2X_2_/_3_ receptors as a possible signaling pathway (44).

LL37 is a unique cathelicidin expressed in humans with potential activity at the P2X_7_ receptor (45). In this context, Chotjumlong and colleagues demonstrated that LL-37 can increase COX-2 and PGE2 expression in a dose- and time-dependent manner through the activation of MAPK P2X_7_/ERK and p46 JNK receptors in human gingival fibroblasts (HGFs) isolated from gingival biopsies. Furthermore, this mechanism may promote the proinflammatory effects of LL-37/P2X_7_ in oral mucosal inflammation associated with periodontal diseases (46). MAPK activity is required for cytosolic phospholipase A_2_ activity (47, 48). Activation of the P2X_4_ receptor triggers calcium influx and p38 MAPK phosphorylation, leading to cytosolic PLA_2_ (cPLA_2_) activation and COX-dependent PGE2 release. However, P2X_4_R-deficient mice exhibit impaired PGE2 production and reduced inflammatory pain behaviors (49). In mast cells, degranulation is mediated by the synergistic action of P2X_4_ and PGE2 via EP3 receptors, which induce Ca2+ influx in a Ca2+-dependent manner (50). This costimulation of P2X_4_ and PGE2 receptors led to increased expression of cytokines, including TNF-α, IL-6, and IL-13, via activation of the nuclear factor κB pathway (51). On the other hand, the interaction between prostanoids and P2X receptors indicates that PGE2 can inhibit the increase in [Ca2+]i induced by 10 μM ATP in J774 macrophages after they are sensitized to ivermectin. Ivermectin is an allosteric modulator of the P2X_4_ receptor that can potentiate ATP-induced Ca2+ responses (52). These data suggest that PGE2 may inhibit P2X_4_ activity in these cells (53).

On the other hand, studies have shown that P2X_7_ inhibition can decrease prostaglandin release. Li *et al.* demonstrated that fluid shear stress (FHS) stimulation results in PGE2 release in MC3T3-E1 osteoblasts and MLO-Y4 osteocytes; this effect is suppressed after the application of Brilliant Blue G (BBG), a nonselective inhibitor of the P2X_7_ receptor. These authors reported that ATP signaling via the P2X_7_ receptor is necessary for prostaglandin release by bone cells and for osteogenesis (54). Similarly, P2X_7_ receptor activation can promote increased IL-1β and PGE2 release in peritoneal macrophages and blood monocytes stimulated with LPS and ATP. Consequently, this process leads to fever in mice, an effect that is neutralized by deletion of the P2X_7_ gene or by treatment with the selective antagonist A438079 (55). Thus, the P2X_7_ receptor antagonists BBG and A438079 attenuate PGE2 release through pharmacological inhibition of the receptor.

### Leukotrienes

Leukotrienes are proinflammatory lipid mediators derived from arachidonic acid (AA) via the 5-lipoxygenase (5-LOX) pathway and are important modulators of the inflammatory response. They are produced mainly by immune cells, including neutrophils, eosinophils, mast cells, and macrophages. The translocation of 5-LOX to the nuclear membrane, associated with the activity of its activating protein FLAP, results in the formation of the intermediate LTA_4_, which is converted to leukotriene B_4_ (LTB_4_) or cysteinyl leukotrienes (LTC_4_, LTD_4_, and LTE_4_). LTB_4_ acts as a potent chemotactic agent and leukocyte activator, promoting cell migration, endothelial adhesion and the production of reactive oxygen species, whereas cysteinyl leukotrienes play prominent roles in allergic inflammatory responses, bronchoconstriction processes and increased vascular permeability. The activation of the leukotriene pathway depends on initial events, including increased intracellular Ca^2+^, the activation of phospholipase A_2_ and the subcellular translocation of 5-LOX, and processes can be modulated by extracellular signals such as ATP. Additionally, recent studies highlight the role of these mediators not only in respiratory diseases, such as asthma but also in systemic inflammation and in different tissues, reflecting their importance in diverse inflammatory responses (56, 57). P2X receptors play important roles in the activation of intracellular signaling cascades, leading to the production of inflammatory mediators. In this context, the functional interaction between purinergic signaling and leukotriene biosynthesis is relevant to inflammation (58).

Evidence of the connection between P2X receptors and leukotrienes has recently been described in studies using experimental models of the central nervous system. P2X_7_ receptor activation in microglia induces an increase in intracellular Ca^2+^ and the activation of Ca^2+^/calmodulin-dependent protein kinase II (CaMKII), a pathway that promotes the translocation of 5-LOX, an event identified as a critical step toward increasing leukotriene production in a model of subarachnoid hemorrhage. The authors demonstrated that this signaling contributes to microglial dysfunction and the perpetuation of neuroinflammation by establishing a connection between purinergic activation and the regulation of not only the production but also the subcellular localization of the enzymatic machinery responsible for leukotriene synthesis (59). Similarly, in cells of the peripheral immune system, such as neutrophils, purinergic signaling has been associated with the amplification of leukotriene biosynthesis. Extracellular ATP associated with formylated peptides potentiates LTB_4_ production through the induction of sustained Ca^2+^ flow, promoting the recruitment and amplification of neutrophils to the inflammatory site. A phenomenon characterized as *neutrophil swarming*, which is a process that occurs in the context of tissue damage or pathogenic invasion and contributes to the local amplification of the inflammatory response (60). Although the study did not identify a single P2X receptor subtype exclusively responsible for this effect, the predominant expression of P2X_1_ and P2X_4_ in neutrophils suggests a significant role for these receptors in the upstream modulation of leukotriene synthesis (61, 62).

The role of cysteinyl leukotrienes in inflammatory processes associated with infections has received increasing attention. In an experimental model of cutaneous leishmaniasis, the production of cysteinyl leukotrienes was related to a protective effect, favoring the control of infection by strengthening the immune response against the parasite, and this effect seems to result from the activation and recruitment of immunocompetent cells essential for the containment of the infectious process (63). Although the study focused predominantly on the biological consequences of the presence of leukotrienes without delving into the cellular mechanisms underlying their biosynthesis, the results indicate that the release of these mediators is a determinant of immune response efficacy, regardless of the pathways responsible for their generation. In this context, although purinergic signaling has not been directly examined, the release of extracellular ATP in infected and inflamed tissues supports the hypothesis that purinergic receptors, including P2X receptors, can indirectly influence both the production and action of cysteinyl leukotrienes (64). In addition to the P2X_7_ receptor, other P2XR subtypes, particularly P2X_4_, which is widely expressed in glial cells, are associated with neuroinflammatory responses and serve as key markers. In addition, studies carried out by Salcman *et al.* (2021) revealed how the activation of different subtypes of P2X receptors modulates the activity of mast cells and glial cells, influencing the intensity and duration of the inflammatory response; however, the direct interaction between these receptors and the leukotriene pathway, the ability of these channels to promote the influx of Ca^2+^, and the regulation of important cells involved in the biosynthesis of leukotrienes suggest a modulatory role in this scenario. Although specific studies have not directly addressed the induction of leukotrienes by P2X_4_Rs, evidence has shown that this receptor regulates the production of other eicosanoids, such as prostaglandins, suggesting P2X_4_-mediated signaling in inflammatory lipid pathways (49). Thus, the absence of direct data does not invalidate their pathophysiological relevance but highlights an important gap in the literature that warrants investigation. In this context, it is possible to observe that the association between leukotrienes and P2X_4_ occurs through inflammatory pathways, considering that this lipid is associated with neutrophil recruitment and cell permeabilization, assisting in an inflammatory feedback process and promoting the release of ATP, which leads to the activation of P2X_4_.

The activity of the P2X_1_ receptor, in turn, is associated with the activation of immune cells and platelets, promoting the activation of phospholipase A_2_ and the release of arachidonic acid, demonstrating the role of this receptor in the formation of proinflammatory lipid mediators, including leukotrienes, especially in acute inflammatory and thrombo-inflammatory contexts (61).

### Thromboxanes

Thromboxanes are proinflammatory lipids present mainly in platelets. However, these lipids can be released by other cell types, such as macrophages, which are immune cells present during the inflammatory process, and endothelial cells, which line the inner surface of blood vessels (65, 66). Thromboxane production occurs when a cell is activated during the inflammatory process or during injury; in this context, the enzyme phospholipase A_2_ releases arachidonic acid from membrane phospholipids, and this arachidonic acid is then converted into prostaglandin H2 (PGH2) by enzymes of the cyclooxygenase pathway, with this enzyme thromboxane-A synthase, converting PGH2 into thromboxane A_2_ (67).

Another mechanism of thromboxane activation may involve ATP release via Toll-like receptor 2 (TLR_2_) stimulation. This is because ATP activates the P2X_1_ receptor, which is involved in regulating platelet function. The P2X_1_ receptor activates p38 MAPK, which can increase platelet secretion and aggregation when thromboxane A_2_ levels are low, generating a positive feedback loop via P2X_1_ receptor activation (68, 69). The importance of MAPK p38 kinase activation was demonstrated by Huang and collaborators, who reported that P2X_1_ stimulation activates the P38 pathway, thereby increasing platelet secretion. The same study revealed that inhibition of P38 MAPK blocked platelet aggregation, demonstrating the influence of the P2X_1_ receptor on the amplification of the thromboxane A_2_ response (70). Additionally, P2X_1_ overexpression can potentiate thromboxane signaling, which can increase Ca^2+^ influx and platelet activation. Although P2X_1_ cannot activate platelet aggregation on its own, it amplifies the effects of thromboxanes, collagen, and ADP via P2Y_12_ (71).

### Specialized proresolving mediators (SPM)

The specialized mediators for the resolution of inflammation are bioactive lipids derived from omega-3 fatty acids. They act by reducing inflammation and restoring homeostasis in the body. SPMs can be divided into lipoxins, resolvins, protectins, and machines (72). This group acts by modulating the inflammatory process through receptor pathways, including the P2X family of receptors. Notably, SPMs target GPR18, a member of the G protein-coupled receptor (GPCR) family. This receptor is involved in immune regulation. SPMs that act on GPR18 inhibit the P2X_3_ receptor by blocking the AC/cAMP/PKA signaling pathway (73). Previously, the administration of R1 maresins in mice reduced NF-κB production, with subsequent decreases in TNF-α and IL-1β levels and inflammation and pain (74). This same work revealed that maresina R1 can inhibit carrageenan-induced neutrophil and macrophage recruitment. This decrease in TNF-α and IL-1β levels may reduce P2X_7_ receptor activation because, when activated, IL-1β stimulates ATP release, which promotes P2X_7_ receptor activation and enhances the inflammatory effect (75). Protectins act by reducing the inflammatory process. In addition, protectins act by decreasing cytokine production and leukocyte migration, indicating that this bioactive lipid not only exerts anti-inflammatory effects but also contributes to homeostatic mechanisms (76).

### Lipoxins

Lipoxins are a class of endogenous lipid mediators derived from arachidonic acid, especially lipoxin A_4_ (LXA_4_). Unlike leukotrienes, which amplify and sustain the inflammatory response, lipoxins play a central role in the inflammation resolution phase and act by reducing neutrophil recruitment and activation, promoting the phagocytosis of apoptotic cells by macrophages, and negatively modulating the production of proinflammatory cytokines. The physiological relevance of lipoxins has been demonstrated in several contexts, including lung inflammation, cardiovascular diseases, sterile inflammation, and chronic inflammatory processes associated with persistent tissue damage (77, 78).

Lipoxins are formed via transcellular pathways involving the action of enzymes such as 5-LOX, 12-LOX, and 15-LOX from the release of AA. The biological action of lipoxins is associated with the activation of the FPR2/ALX receptor, a G protein-coupled receptor widely expressed in cells of the immune system, and this metabotropic receptor modulates the action of lipoxins in association with other signaling pathways related to inflammation, such as ATP-mediated purinergic signaling (79, 80, 81).

In this context, experimental evidence indicates that purinergic signaling modulates steps in lipoxin synthesis, highlighting the activation of the P2X_7_ receptor in immune cells. *In vitro* research has indicated that sequential stimulation of pattern recognition receptors, such as TLR_4_, followed by activation of P2X_7_ with ATP leads to the hydrolysis of 15-HETE by cPLA_2_ and the subsequent release of this precursor for conversion to bioactive lipoxins. Although P2X_7_ is widely recognized for its participation in the activation of the NLRP3 inflammasome and in the release of proinflammatory cytokines, these findings indicate that P2X_7_ receptor activation contributes to the reorganization of cellular lipid metabolism. The immune cells involved in this process can store lipid precursors and release them after stimulation, creating biochemical conditions that favor the generation of proresolutive mediators and the transition to the resolution phase of inflammation (82).

More recent evidence from human cell models has demonstrated a direct association between P2X_7_R activation and lipoxin production. Macrophages derived from individuals with asthma stimulated with Bz-ATP, a selective receptor agonist, significantly increased the levels of lipoxin A_4_ (LXA_4_) and other proresolutive mediators, such as resolvin D1. In contrast, pharmacological inhibition of the receptor via the antagonist AZD9056 promoted a reduction in the expression of these mediators and confirmed the direct participation of P2X_7_ in the biosynthetic production of lipoxins. These findings are particularly relevant because they involve human cells directly involved in chronic inflammatory diseases, providing consistent functional evidence of the connection between purinergic signaling and the generation of resolutive mediators. These results are of paramount importance since they use human cells directly involved in chronic inflammatory pathologies. Thus, solid functional evidence has established a link between purinergic signaling and the production of resolution mediators (83).

Other studies have shown that lipoxin A_4_ also acts directly on inflammatory pathways associated with the NLRP3 inflammasome. In models of monosodium urate (MSU) crystal-induced inflammation, LXA_4_ reduced NLRP3 inflammasome activation, thereby decreasing caspase-1 cleavage and IL-1β release in rats with gouty arthritis. These findings are particularly relevant because NLRP3 is involved in key outcomes of P2X_7_ receptor signaling across diverse inflammatory conditions. Thus, in the inhibition of inflammasome activation, LXA_4_ can regulate inflammatory responses initially amplified by purinergic signaling, contributing to the transition from inflammation to the resolution phase (84).

### Resolvins

Resolvins are proinflammatory lipids derived from fatty acids; they are classified into two series: E-series resolvins (RvEs) and D-series resolvins (RvDs). Rhea-N is a lipid derived from eicosapentaenoic acid (EPA). The main function of these lipids is to terminate the inflammatory process by regulating neutrophil recruitment to the site of inflammation, reducing the production of proinflammatory cytokines, and stimulating tissue regeneration (85). On the other hand, RVs are bioactive lipids derived from docosahexaenoic acid (DHA). This type of lipid is widely found in the cell membrane and in the brain. RVd function is similar to that of RVEs, but this class of resolvins is notable for protecting neurons and promoting recovery from brain injury or neurodegeneration (86).

Some studies have reported that in patients with Alzheimer's disease, the levels of bioactive lipids, such as resolvins, are reduced, whereas the levels of prostaglandin D2 are elevated. Another study reported that the administration of RvE and RvD resolvins, along with other bioactive lipids, and the consequent control of microglial activation improved memory in mice with Alzheimer's disease (87, 88). In addition, after being incubated with lipopolysaccharide, microglia treated with resolvins presented decreased levels of proinflammatory cytokines and reduced NF-κB activation (89).

Resolvins can also act through purinergic receptors, including members of the P2X family. A study conducted in 2023 revealed that alveolar macrophages from people with asthma treated with Bz-ATP, an already well-described agonist of the P2X_7_ receptor, presented increased levels of resolvin D1. The presence of the antagonist AZD9056 reversed this effect, suggesting a correlation between pore activation and the level of resolvin D1 (82). In addition to P2X_7_, resolvins also act on the P2X_3_ receptor because D2 resolvins can inhibit P2X_3_ pore functionality via activation of GPR18, a G protein receptor that plays an important role in the regulation of the nervous system and inflammatory response (90). This indirect modulatory effect of D2 resolvins on the functionality of the P2X_3_ pore was well elucidated by the work of Hao and collaborators, who demonstrated that RvD2 reduces the amplitude of currents induced by α,β-methylene-ATP, an agonist of the P2X_3_ receptor, in primary sensory neurons. Additionally, when an animal was treated with RvD2 before the administration of α-methylene-ATP, pain symptoms were reduced (91). These data indicate that resolvins modulate purinergic receptors during the inflammatory process.

### Cholesterol

Cholesterol is a lipid that is fundamental to the structure of the plasma membrane. This lipid acts by regulating the fluidity and organization of the membrane and serves as a precursor for steroid hormones, bile acids, and vitamin D. Cholesterol can modulate the inflammatory process and cell signaling through the formation of lipid rafts (92, 93). Lipid rafts are microdomains of the membrane that are rich in sphingolipids, cholesterol, and specific proteins. They play important roles in the organization of the plasma membrane, assisting in cell signaling, protein trafficking, and cell‒cell interactions. These microdomains are associated with several pathological processes, including neurodegenerative diseases, viral infections, and cancer (94).

Some studies have reported that certain P2X subtypes, mainly P2X_4_ and P2X_7_ receptors, are present in lipid rafts. A study published by Murrell-Lagnado reported that the cholesterol present in the membrane can modulate P2X receptor signaling. He reported that the P2X_1_ receptor is highly dependent on cholesterol to maintain the correct channel conformation and that cholesterol depletion reduces P2X_1_ ion currents (95). These data reinforce the findings of a 2006 study showing that depletion of membrane cholesterol reduces P2X_1_ receptor-mediated Ca^2+^ influx by approximately 80%. Additionally, P2X_1_ is highly expressed in cholesterol-rich regions of the membrane (96), and cholesterol interacts with the transmembrane domains of the receptor, contributing to its structural stability (97).

Unlike P2X_1_ receptors, P2X_2_ and P2X_3_ receptors are less dependent on cholesterol because their depletion does not alter P2X_2_ currents. Thus, cholesterol is more strongly associated with a structural function than with a modulatory function of P2X_2_, increasing the rigidity and organization of the lipid bilayer (98). In contrast, cholesterol-derived bile acids are capable of inhibiting the P2X_2_ receptor in rats, but they do not significantly inhibit the P2X_1_, P2X_3_, P2X_4_, or P2X_7_ receptor (99). P2X_3_ is generally found in cholesterol-rich regions of the membrane and is essential for functional stability. The removal of cholesterol in these regions can disrupt membrane microdomains, reducing the amplitudes of ATP-induced currents and altering the desensitization of the P2X_3_ receptor. Thus, cholesterol indirectly modulates the P2X_3_ receptor by maintaining membrane lipid rafts, which are essential for receptor stability. When cholesterol is removed from lipid rafts, P2X_3_ migrates to nonraft regions that are poor in cholesterol, leaving the membrane more fluid and disorganized and decreasing the responsiveness of the receptor to ATP (100, 101).

P2X_4_ is among the main purinergic receptors regulated by membrane cholesterol. High cholesterol levels inhibit the opening of P2X_4_ receptor pores, but P2X_4_-induced currents increase at low cholesterol levels. Barraco *et al.* reported that cholesterol metabolites can modulate ATP-induced ion currents in P2X_4_ and P2X_7_ receptors in microglia. This study also suggested that this negative modulation of the P2X_4_ receptor occurs because cholesterol alters the composition of the plasma membrane, altering its fluidity, thickness, and microdomains and influencing the opening and kinetics of the P2X_4_ receptor channel (102). Another way that bioactive lipids can modulate P2X_4_ is through some cholesterol-derived bile acids, such as lithocholic acid (LCA). A 2020 study revealed that this lipid is capable of inhibiting the opening of the P2X_2_ channel, but it potentiates the opening of the channel and the ion currents of the P2X_4_ receptor. Interestingly, this potentiation of the receptor occurs because the lipid molecule binds to an allosteric site on the receptor and does not compete with the physiological agonist ATP, which binds to the target site of P2X_4_ (103, 104).

P2X_7_ is the main receptor of the P2X family affected by cholesterol. This receptor is highly dependent on membrane cholesterol because its full function depends on the presence of cholesterol in lipid rafts. Cholesterol negatively modulates the P2X_7_ receptor, hindering the opening of its pore and the formation of high-conductance pores, which are stimulated by prolonged exposure to the agonist ATP. Studies have shown that P2X_7_ activation is increased in cells treated with methyl-β-cyclodextrin, an oligosaccharide responsible for removing cholesterol from the plasma membrane, indicating that cholesterol is capable of attenuating receptor activation (105). In general, excess membrane cholesterol is detrimental to the activation of the immune system, given that controlled activation of P2X_7_ is essential for maintaining the body's balance. This can be supported by the work of Hutteau-Hamel and colleagues. This study revealed that a diet rich in fats, such as cholesterol, negatively regulates the function of the P2X_7_ receptor in the CD4^+^ and CD8^+^ T cells of C57BL/6J mice. As a result, the body is unable to respond efficiently to infections and control the inflammatory process, facilitating the onset of disease (106).

### Ceramides

Like cholesterol, ceramides are associated with membrane organization and cell signaling functions. In the context of cell signaling, this bioactive lipid is linked mainly to the processes of modulating apoptosis and the response to cell stress and inflammation. Excess ceramides can cause inflammation through cell stress. Ueda reported that ceramides induce apoptosis in renal cells, and this work revealed that this apoptotic effect occurred mainly in mitochondria. This is because ceramides cause the activation of caspases and the release of cytochrome C, triggering a process of cell death by apoptosis (107, 108). Experimental evidence has shown that activation of the P2X_7_ receptor by extracellular ATP can induce apoptosis in thymocytes, triggering de novo synthesis of ceramide, independent of the sphingomyelinase pathway, which makes this sphingolipid a central mediator in the apoptotic process. ATP-induced apoptosis is directly linked to mitochondrial dysfunction, as evidenced by the loss of mitochondrial membrane potential and subsequent activation of caspase-9 and caspase-3. These data corroborate the central role of ceramide as a mediator that connects P2X_7_ signaling to the induction of programmed cell death in thymocytes (109). Activation of the P2X_7_ receptor can induce membrane lipid remodeling, which favors the generation of ceramides. Ceramide generation is not exclusively dependent on P2X_7_ receptor activation, but during the inflammatory process, the P2X_7_ receptor binds to the enzyme n-SMase, leading to ceramide activation (110).

Ceramides also play a role in the immune system, as they can activate immune cells such as macrophages and dendritic cells. During inflammation, immune stimuli such as cytokines and antigens generate ceramides through the hydrolysis of sphingomyelin by sphingomyelinases. In this context, ceramides act as lipid messengers for immunosuppressive and pro-apoptotic signals, suppressing the inflammatory response (111). Studies have indicated that the activation of the purinergic P2X_7_ receptor by ATP has a modulatory effect on the lipid metabolism of immune cells, including ceramide synthesis, influencing proapoptotic pathways and regulating the inflammatory response (112). Additionally, Raymond and LeStunff investigated how the de novo biosynthesis of ceramide contributes to macrophage death after P2X_7_ receptor activation. The exposure of macrophages to ATP or the selective agonist BzATP promoted an increase in intracellular ceramide levels, and inhibition of the de novo synthesis pathway significantly reduced the accumulation of this lipid. In addition, ceramide generation has been shown to be essential for ATP-induced caspase-3 and caspase-7 activation, corroborating the link between P2X_7_-dependent cell death and the presence of this bioactive lipid, which influences critical cellular processes in the immune system (113).

These findings suggest that ceramide functions as a central lipid messenger capable of integrating purinergic signals via P2X_7_ and modulating both programmed cell death and the immune response in different cell types. In this context, the data reinforce that the interaction of ceramide with the CD300f receptor in mast cells prevents ATP-induced activation, reducing inflammation in colitis models and confirming that, in addition to the participation of lipids in the induction of cell death, ceramide plays an important anti-inflammatory regulatory role by integrating purinergic signaling with the maintenance of immune homeostasis (114).

## Discussion

In summary, the data presented in this review demonstrate that bioactive lipids and other lipid mediators interact with P2X purinergic receptors, thereby modulating key cellular events. Table 1 offers an integrated view of the interconnection between lipid and purinergic signaling via P2X, summarizing the interplay between lipids and their target receptors in the regulation of inflammatory processes.

**Table 1:** Modulation between P2X receptors and bioactive lipids

Lipids	Target Receptor	Mechanism of Action	References
Lysophosphatidic acid	P2X	LPA can induce ATP release in rat microglia cultures via the LPA3 receptor, inducing activation of P2X receptors through ATP released by the cells.	31
	P2X_3_	LPA increases the functional activity of α, β-methylene-ATP at P2X_3_ receptors in dorsal root ganglion (DRG) neurons via LPA1 receptors, Gq/11 proteins, and the PKC signaling pathway.	35, 36
	P2X_7_	Activation of the P2X_7_ receptor by ATP stimulates the production of phospholipase D and A_2_. This stimulation generates LPA, which promotes the formation of membrane vesicles via Rho-associated kinase in osteoblasts.	32, 33
Prostaglandins	P2X_3_	PGE2 binds to the EP3 receptor, thus activating the cAMP/PKA signaling cascade, leading to increased P2X_3_ expression.	42, 43
	P2X_4_	Activation of the P2X_4_ receptor can lead to increased production of PGE2 via phospholipase A_2_, resulting from increased Ca^2+^.	49–53
	P2X_7_	Activation of the P2X_7_ receptor can release PGE2 via membrane lipids.	54, 55
Leukotrienes	P2X_7_	The increase in Ca^2+^, through the activation of the P2X_7_ receptor, can lead to the activation of CaMKII, resulting in the translocation of 5-LOX.	59
	P2X_1_	Activation of the P2X_1_ receptor in immune cells promotes the activation of phospholipase A_2_ and the release of arachidonic acid, which can be converted into leukotrienes via the lipoxygenase pathway.	61, 62
	P2X_4_	The release of leukotrienes during an inflammatory process helps activate the	65
		P2X_4_ receptor due to the release of ATP by immune cells.	
Thromboxanes	P2X_1_	Activation of the P2X_1_ receptor can enhance thromboxane A_2_ production through activation of the p38 MAPK kinase, increasing secretion and platelet aggregation.	69, 70
Lipoxins	P2X_7_	Activation of the P2X_7_ receptor can lead to increased lipoxins in asthma patients through cPLA_2_ activation.	83, 84
Resolvins	P2X_3_	D2 resolvins can inhibit P2X_3_ pore functionality via activation of GPR18	91
	P2X_7_	Activation of the P2X_7_ receptor can lead to increased levels of RvD1 in alveolar macrophages.	83
Cholesterol	P2X_1_	Cholesterol, present in the cell membrane, has been shown to be essential for the stability of the P2X_1_ receptor.	96
	P2X_2_	The cholesterol present in the cell membrane is related to the functionality of the P2X_2_ receptor and can also inhibit the opening of its channel.	99, 100, 104
	P2X_3_	Cholesterol, present in the cell membrane, has been shown to be essential for the stability of the P2X_3_ receptor.	101, 102
	P2X_4_	Cholesterol present in the cell membrane can enhance the opening of the channel and the ionic currents of the P2X_4_ receptor.	104, 105
	P2X_7_	Cholesterol present in the cell membrane can reduce the opening of the P2X_7_ receptor channel when at high levels.	106
Ceramide	P2X_7_	Activation of the P2X_7_ receptor stimulates the formation of new ceramides in the cell membrane.	110–114

A set of metabolic pathways forms an integrated system to produce bioactive lipids derived from polyunsaturated fatty acids (PUFAs), and the balance between proinflammatory and proresolving mediators is crucial for the progression or resolution of the inflammatory response (18). Data from the literature indicate that proinflammatory lipids indirectly mediate the expression of P2X receptors. This occurs because proinflammatory lipids increase ATP release via the PKA, PKC, and MAPK pathways. Furthermore, P2X receptors, such as P2X_7_, can mediate the release of lipids of this class, such as prostaglandins and lysophosphatidic acid, increasing Ca^2+^ influx, which leads to the activation of phospholipase A_2_. Arachidonic acid products can also modulate cAMP signaling. Okonogi *et al.* reported that prostaglandin E2 increases cAMP levels in macrophages. This occurred after murine peritoneal macrophages were treated with 100 nM PGE2. This occurs because PGE2 binds to EP2/EP4-type G protein-coupled receptors, leading to the activation of the adenylate cyclase enzyme, which is responsible for the conversion of ATP to cAMP. As is known in the literature, increased cAMP increases PKA activity (115, 116).

A 2018 study demonstrated that electroacupuncture had an analgesic effect on rats through the downregulation of P2X_3_ receptors in the dorsal root ganglia after PKC suppression. This occurs because PKC does not act directly on the P2X_3_ receptor but rather in the process of sensitizing the receptor, intensifying its response to ATP and neuronal signals related to pain and thus contributing to pain hypersensitivity (117, 118). As previously described, phospholipase A_2_ can activate PKC through Ca^2+^ and arachidonic acid, which favors the formation of diacylglycerol, as demonstrated in van Rossum's work. In this work, he proposed that PLA_2_ and PKC interact and regulate each other through an integrated Ca^2+^ pathway (36, 119). Furthermore, another way to activate PKC involves the activity of histamine H_1_ receptors. Histamine is a proinflammatory mediator that can be released through activation of the P2X_7_ receptor (120). Another modulation that can occur between lipids and P2X receptors is the potentiation of thromboxane A_2_ production through activation of the P2X_1_ receptor. This occurs because when the P2X_1_ receptor is activated by ATP, it increases the influx of Ca^2+^, activating the phospholipase A_2_ pathway, which releases arachidonic acid into the membrane, where it is converted into thromboxane A_2_ by COX-1 (68).

However, bioactive lipids can also promote the resolution of inflammation and negatively modulate P2X receptors because some lipids, such as resolving resolvins, can reduce the release of proinflammatory cytokines. SPMs also promote ATP degradation, leading to reduced P2X expression, particularly that of P2X_7_, which is the main P2X receptor involved in the inflammatory process (75). In addition to modulating P2X receptors, bioactive lipids are essential for the structural organization and proper function of these receptors.

## Conclusion

The data from this review indicate that the signaling cascade mediated by bioactive lipids directly and indirectly modulates P2X purinergic receptor signaling, both positively and negatively, as shown in Table 1. Activation of phospholipase A_2_ is extremely necessary for the release of proinflammatory lipids via P2X receptors since its activation is associated with the release of lysophosphatidic acid, prostaglandins, leukotrienes, and thromboxanes. Calcium influx is an indirect pathway through which P2X receptors activate phospholipase A_2_. In this context, we can conclude that the activation of P2X receptors is among the main pathways involved in the increased production of proinflammatory lipids. Interestingly, the P2X_7_ receptor can indirectly activate the P2X_3_ receptor, as it increases Ca^2+^ influx, which triggers phospholipase A_2_ activity and initiates a signaling cascade that leads to PKA activity, a pathway responsible for increased P2X_3_ expression. Furthermore, PKC activation can intensify the P2X_3_ receptor response to ATP. On the other hand, lipids act by reducing the inflammatory process; they inhibit cytokine production and reduce leukocyte migration, in addition to degrading ATP, ADP, and AMP into adenosine, drastically reducing the activation of P2X receptors, especially P2X_7_, which requires higher concentrations of ATP to function fully.

**Fig. 1:**
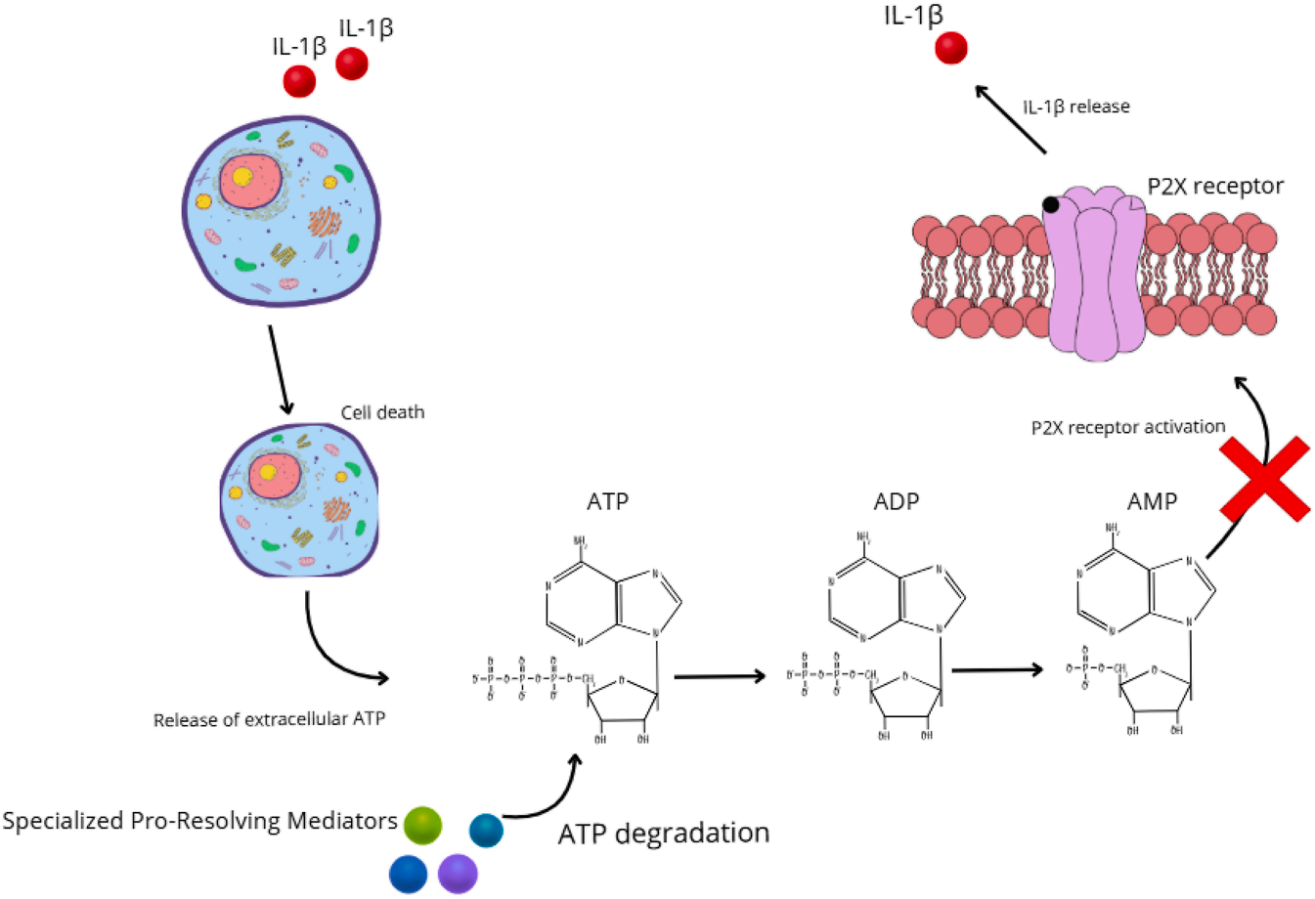
Schematic representation of the mechanism by which specialized proresolving mediators negatively modulate purinergic signaling. Cell death leads to the release of extracellular ATP, which activates P2X receptors and promotes the release of IL-1β. Specialized proresolving mediators increase the degradation of extracellular ATP into ADP and AMP, reducing the activation of P2X receptors and, consequently, the release of IL-1β, thus contributing to the resolution of inflammation.

## Data availability

The authors of this work are committed to submit the data requested by the reviewers.

## Conflict of interest

The authors declare that they have no conflicts of interest with the contents of this article.
